# Correction to: Struggle in the bubble - a prospective study on the effect of remote learning and distance education on confidence in practical surgical skills acquired during COVID-19

**DOI:** 10.1186/s12909-023-04195-3

**Published:** 2023-04-03

**Authors:** Felicia Kneifel, Haluk Morgul, Shadi Katou, Jens P. Hölzen, Benjamin Strücker, Mazen Juratli, Andreas Pascher, Felix Becker

**Affiliations:** Department of General, Visceral and Transplant Surgery, University Hospital Münster, Münster, Germany

**Correction: BMC Medical Education (2023) 23:115** 10.1186/s12909-023-04092-9

Following publication of the original article [1], we have been informed that due to a typesetting error, the author name of reference 22 was incorrectly cited.

Incorrect: Al SA. The impact of the COVID-19 pandemic on medical education. Br J Hosp Med (Lond). 2020;81(7):1–4.

Correct: Al Samaraee A The impact of the COVID-19 pandemic on medical education. Br J Hosp Med (Lond). 2020;81(7):1–4.

The original article has been corrected.

The publishers apologise for this error.
